# Medicine

**Published:** 1915-03

**Authors:** J. R. Charles


### Progress of the Medical Sciences.

### MEDICINE.

**The Colloidal Gold Reaction in the Cerebro-Spinal Fluid.**—Lee and Hinton[1] form a very favourable opinion of the value of this test, originally described by Lange, in syphilitic conditions of the nervous system. The theory of the reaction is based on the following observations: (1) Solutions of electrolytes precipitate colloidal gold; (2) proteins in the absence of an electrolyte also precipitate a solution of colloidal gold; (3) proteins in the presence of an electrolyte inhibit precipitation in colloidal gold solutions.

Lange's theory is (1) that substances in pathological spinal fluids will precipitate colloidal gold, provided the globulin and nucleoprotein fractions are held in solution with a 0.4 per cent. sodium chloride solution, and (2) that there is a characteristic change for certain diseases involving the central nervous system. The technique is fairly simple, and is described in detail in this paper, and also in another communication by Miller and Levy.[2]

Lee and Hinton investigated 100 consecutive cases, and compared the results they obtained by this method with the clinical diagnosis and other laboratory tests. They found that the gold reaction was not always parallel with the blood Wassermann reaction, the spinal fluid Wassermann reaction, the globulin test, or the cell count, but that it was apparently more constant in syphilitic affections than any of the other tests. The gold reaction typical of syphilis occurred only twice in the absence of blood Wassermann reaction, spinal fluid Wassermann reaction, cell counts, and globulin tests. In both these cases the gold reaction was confirmed by clinical diagnosis. The gold reaction usually occurs, in combination with a positive finding in one of the other tests, but there is no constant association between this test and any one of the other tests. They found the blood Wassermann reaction present in only 43 per cent. of the cases diagnosed as syphilis of the central nervous system, and both blood Wassermann reaction and spinal fluid Wassermann reaction absent in 24 per cent. of the cases of presumable syphilis of the central nervous system. A negative gold reaction was obtained in two cases diagnosed as syphilitic. One of the advantages of this test is the small amount of cerebro-spinal fluid required, viz. 0.2 c.c.

These authors regard the test as more delicate than any of the other laboratory tests, and consider that it is nearly always present in cases of syphilis of the central nervous system. Moreover, the test has the further advantage that it gives reaction with pathological spinal fluids other than those of syphilis, which are characteristic and easily differentiated from that due to syphilis.

Miller and Levy [2] having examined 171 cases by this method, and having also in every case carried out a blood Wassermann reaction, cerebro-spinal fluid Wassermann reaction, cell count and globulin test, reached the following conclusions:—Normal cerebro-spinal fluids give negative reactions. The test can be performed rapidly with a minimum amount of cerebro-spinal fluid. Extreme care is, however, required in the preparation of the reagents. It has no advantage over known laboratory methods in the diagnosis of congenital syphilis. It is of no help in the diagnosis of tubercular and purulent meningitis. Reactions in secondary and tertiary syphilis are inconstant, and their significance when present is not known. The statement that they indicate the earliest stages of involvement of the central nervous system lacks proof. The positive reactions in the majority of cases of tabes and cerebro-spinal syphilis are not characteristic. The authors feel, however, that the reaction peculiar to paresis is sufficiently constant to warrant its use as an aid in the differentiation of this condition from others with which it might be confused. It is possible that the test may prove to be more sensitive than are those at present employed as an indicator of the results of specific therapy in syphilitic disease of the central nervous system. Much may be expected from the application of colloidal chemistry to the study of biological problems.

**The Sympathetic Nervous System and the Gastro-Enteric Functions.**—The influence exerted by the sympathetic nervous system on the functions of the stomach and intestines has been brought into much greater prominence by work done on the sympathetic and autonomic systems during the last ten years. C. S. Fischer [3] says that the great majority of digestive disturbances start as functional defects, many of which exert their influence through the nervous system. Fifty per cent. of the gastric acid secretion is due to psychic-nervous influences, and psychic causes are often the controlling factors in the determination not only of the character but of the organic development of digestive diseases. The physiological and anatomical investigations of Gaskell and Langley have thrown much light on the sympathetic nervous system, while those of Eppinger and Hess have elucidated the pharmacological and clinical aspects. Fischer attempts in his paper to focus attention on these points, and to reduce the facts which are already known to a practical working basis. These facts are of as much importance as the size, shape, position and acidity of the stomach. It should be possible in any given case to determine the reactional trend of the sympathetic nervous system. In a field so comparatively new many problems are, however, still unsolved. The whole sympathetic system is divided into (1) the sympathetic nervous system proper, and (2) the autonomic system. These two systems are antagonistic in their directing influence, and endeavour to maintain a normal balance of function in the structures they control. Good descriptive accounts of the anatomy of these systems, with special reference to points of clinical importance, have recently been published by Fischer,[3] Kinnier Wilson[4] and Elliott[5]. Fischer describes the whole sympathetic structure as a system with four stations: first, the central station, consisting of brain and spinal cord; second, the station consisting of the lateral or vertebral ganglia; third, the station consisting of the collateral ganglia (cervical stellate, epigastric, superior and inferior mesenteric, etc.); fourth, the terminal station in the viscera.

Any impulse starting in the central station may be interrupted and divided either in the lateral or collateral stations. An impulse, however, coming from the periphery may never reach the central station, but may be diverted at the lateral or collateral ganglia, *e.g.* an impulse beginning in the colon and reaching the stomach would pass first to the superior mesenteric ganglion, thence to the epigastric ganglion, and on to the stomach. In this example the lateral ganglia are not affected. The frequency with which colonic affections react on the secretion of the stomach may thus be partially explained, which is of importance in considering the remote effects of chronic appendicitis and ascending colonic stasis. By means of the collateral ganglia all parts of the alimentary canal are so intimately connected that almost any reflex is possible. Impulses from more distant parts must pass by a more circuitous route, and impulses from the gastro-intestinal tract to other organs must pass through two or three stations at least. Thus a reflex from the intestine to the heart must pass *via* the collateral, lateral and stellate ganglia; those from the intestine to the skin (Head's areas) by the collateral and vertebral ganglia. With a working knowledge of these routes, etiologically obscure local abnormalities of function will often be explained by tracing the same to some distant point of unsuspected importance. This is particularly true if there coexists a hyperexcitability of the sympathetic system. Any influence which stimulates the system diminishes the movement and inhibits the secretory activity of the gastro-intestinal tract from the mouth to the anus. If this influence could be complete and uncontrolled, there would result a general paralysis of the tract, without secretion or motion. On the other hand, any influence which leads to depression of the system leads to stimulation of secretory and motor function.

With extreme hyperstimulation of the system there would result a dry mouth with deficient ptyalin activity, paralysis of the muscular structures of the œsophagus. leading to difficult deglutition, impaired passage of food, and idiopathic dilatation. The cardiac orifice of the stomach would be relaxed, leading to rumination and eructation. In the stomach there would be subacidity, achylia, atonic dilatation, with bloating and pressure. The pyloric sphincter would be relaxed, with backward flow of bile and pancreatic secretion. Lack of tone of the common bile duct would lead to constant discharge of bile into the duodenum and urobilinuria. In the small intestine there would be delayed passage of food and delayed digestion, leading to flatulency, deficient absorption and general malnutrition. The large intestine would be atonic, resulting in stasis, fermentation, decomposition, autointoxication, and chronic appendicitis. In the lower portion of the colon fæcal impaction, possibly mucous colitis, rectal pressure and hæmorrhoids. Interference with the return circulation from the bowel would lead to symptoms of head pressure, vertigo and insomnia.

The ability to recognise the possibility of sympathetic influence as an etiological factor will guard against many false diagnoses. Signs and symptoms are present in most cases, though they are generally neglected.

The dominating native stimulant for the sympathetic proper is adrenalin. The cells of the sympathetic ganglia are derived from the same embryological source as the adrenalin-producing cells of the suprarenal capsules (chromaphil tissue). The normal amount of adrenalin in the blood is one to one hundred million parts. Diminution of this leads to muscular weakness, bronzed skin, loss of tone of the blood vessels, contracted pupil. Excess leads to contraction of the blood vessels, increased contractile force of the heart, dilated pupil, decreased gastro-intestinal motility. Extirpation of the pancreas leads to excessive activity of the chromaphil system. Emotional disturbance leads to an excess of adrenalin secretion. According to Elliott,[5] adrenalin cells are present wherever there are blood vessels, and in some examples they seem to usurp entirely the prerogative of the sympathetic nerves. Fighting power, he says, rises with the rise of the blood pressure, reserves of sugar to feed the muscles are hurried up from the liver on the call of the circulating adrenalin, the daily routine of digestion is checked by intestinal inhibition. He further adds that by that curious antithesis of the emotions upon which Charles Darwin laid stress, the machinery employed to prepare for fight may, with the cowardice of civilisation, be set as powerfully in motion only to express fright. And in this the adrenalin is equally or perhaps even more exhausted. The secretory nerves for adrenalin are incorporated in the splanchnics. Hemmeter proposes the hypodermic injection of 0.00015 gram of adrenalin as a test of sympathetic hypersensibility, since this dose will, under such circumstances, produce the same effect as 0.0005 gram in an average case. This test should, however, be used with very great caution, and Loewi's test, which is comparatively safe, should be used instead. If the test is positive, marked mydriasis follows the instillation of one drop of 1 in 1000 adrenalin solution into the eye. When many of the symptoms of excessive sympathetic stimulation are present, the condition is described as sympathicotonia.

Through this system the influence of the emotions plays a most important part in gastro-intestinal diseases. Fischer claims that the great majority of cases in gastrological clinics are indefinite cases with either hyper- or hypo-functional activity secondary to perverted sympathetic influences, though it is often difficult to find where the trouble started. Although there is much evidence that the sympathetic system is connected with the higher cerebral centres by both afferent and efferent fibres, he does not consider that there is any direct relationship between the actual qualitative and quantitative characteristics of the gastric functions in any emotional states.

J. R. CHARLES.

REFERENCES.

1 *Am. J. M. Sc.*, 1914, cxlviii. 33.

2 *Bull. Johns Hopkins Hosp.*, 1914, xxv. 133.

3 *Med. Rec.*, 1915, lxxxvii. 127.

4 *Brit. M. J.*, 1913, i. 1,257.

5 *Ibid.*, 1914, i. 1,393.

